# The Measurement and Analysis of Impedance Response of HeLa Cells to Distinct Chemotherapy Drugs

**DOI:** 10.3390/mi12020202

**Published:** 2021-02-16

**Authors:** Xiangbin Du, Jinlong Kong, Yang Liu, Qianmin Xu, Kaiqun Wang, Di Huang, Yan Wei, Weiyi Chen, Haiyang Mao

**Affiliations:** 1 Department of Biomedical Engineering, Research Center for Nano-biomaterials & Regenerative Medicine, College of Biomedical Engineering, Taiyuan University of Technology, Taiyuan 030024, China; duxiangbin@ime.ac.cn (X.D.); kongjinlong0787@tyut.edu.cn (J.K.); xuqianmin7113@link.tyut.edu.cn (Q.X.); huangjw2067@163.com (D.H.); weiyan@tyut.edu.cn (Y.W.); chenweiyi@tyut.edu.cn (W.C.); 2 Shanxi Key Laboratory of Material Strength & Structural Impact, Institute of Biomedical Engineering, Taiyuan University of Technology, Taiyuan 030024, China; 3 Institute of Microelectronics of Chinese Academy of Sciences, Beijing 100029, China; liuyang6@ime.ac.cn; 4 University of Chinese Academy of Sciences, Beijing 100049, China

**Keywords:** cellular impedance-based sensing, interdigitated electrodes, chemotherapy drug response, cell–electrode resistance, cell–cell resistance

## Abstract

Electric cell–substrate impedance sensing exhibits a real-time and label-free feature to monitor the response of cells stimulated by various biochemical and mechanical signals. Alterations in the currents passing through the cell–electrode system characterize the impedance variations of cells. The impedance responses of HeLa cells under distinct chemotherapy drugs combine the effects of cell proliferation and cell–substrate adhesion. Optimal interdigitated electrodes were selected to explore the impedance responses of HeLa cells. Measurements of impedance of cells in response to three widely used chemotherapy drugs in clinical practice, namely cisplatin, doxorubicin, 5-fluorouracil, were performed. The results demonstrated that distinct impedance responses of HeLa cells to drugs were exhibited and a decrease in measured impedance was observed after drug treatment, accompanied by alterations in the distribution and intensity of the adhesion-related protein vinculin and the rate of cell proliferation. The link between the impedance profiles of HeLa cells and their biological functions was developed based on the circuit model. This study demonstrated the weights of cell proliferation and adhesion of HeLa cells under the treatments of DDP, DOX, and 5-FU, resulted in distinct impedance responses of cells, providing an impedance-based evaluation methodology for cervical cancer treatment.

## 1. Introduction

Electric cell–substrate impedance sensing (ECIS) is a label-free, noninvasive method for monitoring the physiological state of cells in real time by detecting changes in impedance. The electrical properties of cells are regarded as effective markers to explore the complex physiological states of cells [1]. ECIS-based measurements have widely provided real-time and noninvasive biophysical approach to monitor living cells in vitro. The viability of hypoxic cells was regulated by carbonic anhydrase IX inhibitors and well characterized by changes in the impedance of cells [2]. The effect of clinical bacteria isolates on the integrity of epithelial cells was evaluated based on a decrease in the impedance of cells [3]. Moreover, changes in regulatory factors or protein within cells affected cell impedance, for instance, vinculin-null cells exhibited lower cell impedance [4]; Amyloid-β 42 regulates cell adhesion via the TJ protein to regulate impedance of MDCK cells [5]. The ECIS technique provided useful information for estimating the fill factor of muscular stem cells [6]. This technique was also used in previous works to study tumor cell cycle [7], invasion [8], proliferation [9], and the interaction between tumor cells and other stromal cells [10]. Given the significant support of previous studies [4] that showed careful regulation of impedance responses of cells to various stimuli by vinculin-related attachment, in our work, we further combined the effects of cell proliferation and cell adhesion to explore the link between the alterations in impedance response of cells and biological changes of cells using optimized interdigitated electrodes (with the support of advanced simulations).

Numerous studies demonstrated that the impedance of electrodes is determined by the electrical properties of the electrodes, the adhesive degree between cells and the electrodes, and the proliferation of cells on the electrodes [11,12,13]. Low electrode impedance is required to reduce measurement noise, which is regulated by the type of electrode materials, morphology, and the size of the electrodes. Cell-mediated blocking of the area of the electrodes available for current flow may be complex. During the process of proliferation, MCF-7 exhibited a higher impedance response due to closer cell–cell contact [14]. BEAS-2B, A549, NCI-H292, and PBECs cells with different adhesion characteristics cause different impedance changes [15]. Cell adhesion and proliferation are associated with many cell functions and are regulated by various biochemical and mechanical stimulations. However, investigation of the relative weights of these two factors in regulating cell impedance responses is still insufficient. 

Given the significant threat of cervical cancer to women’s health, in our work, the HeLa cell line, a typical cervical cancer cell line, was utilized as an important example of impedance profiles responding to the treatments of three commonly used chemotherapy drugs in clinical practice. In this study, the weighted correlation of cell adhesion and cell proliferation with the impedance responses of HeLa cells treated with dose-dependent cisplatin (DDP), doxorubicin (DOX), and 5-fluorouracil (5-FU) were investigated. It was observed that DDP, DOX, and 5-FU regulated cell proliferation and adhesion via distinct mechanisms [16,17,18]. DDP could inhibit DNA replication, DOX inhibits the activity of topoisomerase II, and 5-FU affects thymine synthase. The different weighted roles of cell proliferation and adhesion due to the treatment with DDP, DOX, and 5-FU [19,20,21] resulted in different impedance responses of HeLa cells under drug treatment. The interdigitated electrodes were designed and fabricated to monitor the impedance of the HeLa cells, and the optimal performance of the electrodes was selected to explore the distinct responses of the HeLa cells treated using different chemotherapy drugs. Drug-mediated cell adhesion and cell proliferation were measured to investigate the drug responses of the HeLa cells. The cell–electrode circuit model was then used to extract the electrical parameters of the HeLa cells to interpret the weighted correlation of cell adhesion and cell proliferation with the impedance responses of the HeLa cells under chemotherapy stimulation. This work demonstrates that the impedance responses of cells could act as a significant biomarker to evaluate the efficacy of chemotherapy drugs, thereby facilitating clinicians to obtain more accurate feedback on patient response to drugs. Additionally, the real-time and label-free impedance tests could shorten the efficacy evaluation cycle of drugs in order to initiate treatment as soon as possible.

## 2. Materials and Methods 

### 2.1. Materials and Reagents

Dulbecco’s modified Eagle medium (DMEM) and fetal bovine serum (FBS) were obtained from Gibco (Carlsbad, CA, USA). Matrigel Matrix was obtained from BD Biosciences (Bedford, MA, USA). CCK-8 was purchased from Dojindo (Kumamoto, Japan). Penicillin and streptomycin were purchased from Thermo Fisher (Waltham, MA, USA). DAPI was obtained from Beyotime (Suzhou, China). Cisplatin (DDP, ≥99%, Sigma, St. Louis, MO, USA), doxorubicin (DOX, ≥99%, Sigma, St. Louis, MO, USA), 5-fluorouracil (5-FU, ≥99%, Sigma, St. Louis, MO, USA), anti-vinculin antibody, and CF 488A-labeled goat anti-mouse IgG secondary antibody were purchased from Sigma (St. Louis, MO, USA). Both the chemical reagents used in the experiments were analytic reagents, and all solvents used in the experiments were deionized water.

### 2.2. Interdigitated Microelectrodes: Fabrication and Simulation

**Figure 1:**
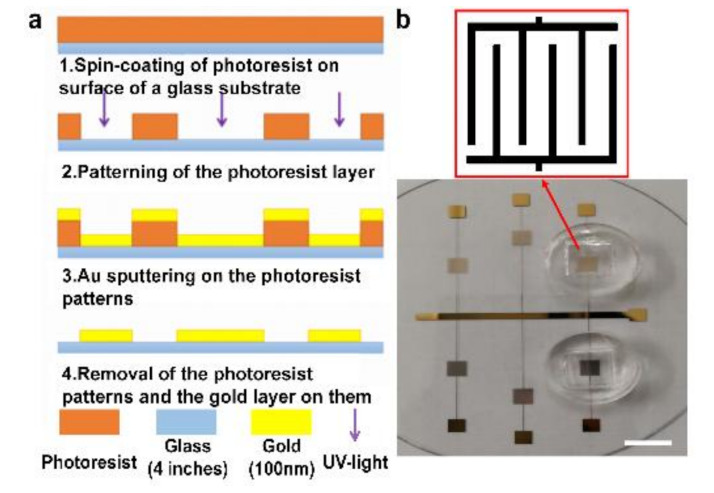
(**a**) Fabrication processes of the electric cell–substrate impedance sensing (ECIS)-based sensor. (**b**) Configuration of the prepared ECIS-based sensor. Scale bar = 1 cm.

The ECIS-based microelectrodes were fabricated by metal lift-off techniques, as shown in Figure 1a. To start the process, AZ6130 photoresist was spin-coated on a glass substrate and then patterned by UV exposure. Then, a layer of Au was sputtered onto the surface of the photoresist. Later, micropatterns of the Au electrodes were formed using the lift-off technique. Finally, a customized PDMS culture well was glued around the microelectrodes. Figure 1b shows the configuration of the electrodes.

**Figure 2:**
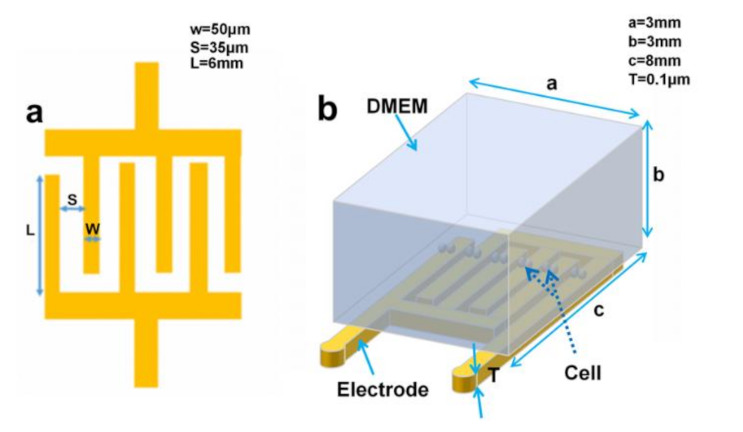
Schematic finite element method (FEM) model of interdigitated microelectrodes, Dulbecco’s modified Eagle medium (DMEM), and cells.

It is essential to optimize structural parameters of the electrodes to obtain the higher sensitivity of monitoring cell status. It was observed that a finger width and finger–finger spacing of the electrodes affected the performance of the electrodes [22,23,24]. In our study, changes in the density of the currents passing through the electrodes were explored before and after addition of cells on the top of electrodes to select the electrodes with the optimal performance. We designed four kinds of interdigitated electrodes (IDEs) with different sizes. Finite element method (FEM) was conducted to investigate the influence of electrode size on the performance of IDEs. The FEM model of the electrodes using DMEM is shown in Figure 2. The model consisted of three components, including the IDEs, the culture medium, and cells, and was simulated using the AC/DC module in COMSOL, with an input voltage of 0.5 V at 40 Hz. The medium was characterized by a solid element, with a conductivity of 1.38 S/m and a relative permittivity of 80, then placed on top of the electrodes. Cells, with a diameter of 15 µm, a conductivity of 0.32 S/m, and a relative permittivity of 5000, were located on the top of the electrodes.

### 2.3. Cell Experiments

#### 2.3.1. Cell Culture

HeLa human cervical carcinoma cells (ATCC number: CCL-2, Gaithersburg, MD, USA) were cultured in DMEM supplemented with 10% FBS, 1% penicillin, and streptomycin, and incubated at 37 °C in a humidified incubator with 5% CO_2_. After 48 h, the HeLa cells were passaged by trypsin, digested, and diluted to appropriate concentrations for subsequent experiments.

#### 2.3.2. Impedance Response Measurement of HeLa Cells

**Figure 3:**
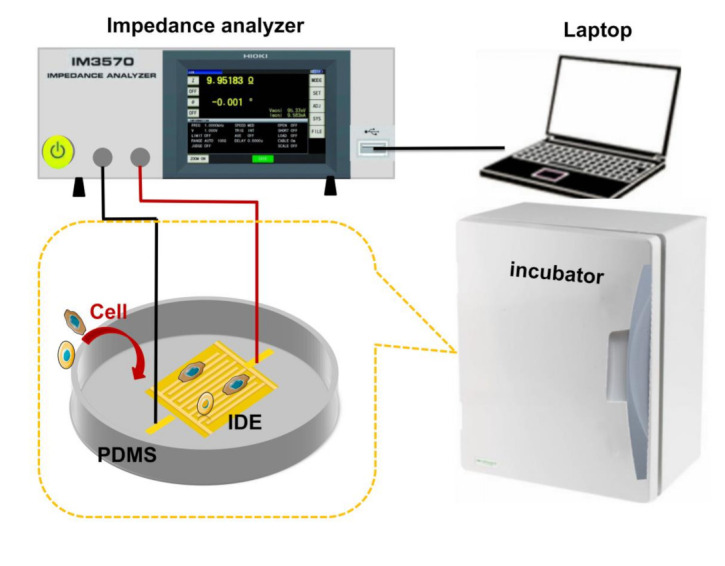
Schematics of impedance measurement of HeLa cells. HeLa cells were placed in the PDMS-enclosed well and then incubated at 37 °C and 5% CO_2_. The impedance of the HeLa cells was monitored in real time.

The coated electrodes were sterilized by soaking them in alcohol under UV light for several hours. Matrigel was diluted with serum-free medium (1:8) and evenly coated onto the surface of the microelectrode substrate to form the gel film. HeLa cells were cultured on the substrate coated with Matrigel at a density of 3 × 10^5^ cells/well for 24 h. The impedance response was taken using an LCR meter (IM3536, HIOKI, Nagano-ken, Japan). The process of impedance measurement is illustrated in Figure 3. The optimal detection frequency of impedance was measured corresponding to the peak value of the normalized impedance, which is defined as follows [22]:

(1) $$Z_{\mathrm{norm}}=\frac{Z_{\mathrm{cell}}\left(f\right)}{Z_{\mathrm{cell}-\mathrm{free}}\left(f\right)}$$

where *Z_cell_* is the total impedance of the cell–electrode system and *Z_cell-free_* is the impedance of the electrodes without cell blocking.

After 24 h of HeLa cell growth on the surface of the microelectrodes, 40 and 80 μg/mL DDP, 40 and 80 μg/mL DOX, and 400 and 800 μg/mL 5-FU were added into culture wells, respectively. The concentrations of DDP, DOX, and 5-FU utilized in the experiment were derived from previous studies [25,26,27]. Drug-treated HeLa cells were continuously incubated for 24 h and untreated HeLa cells were taken as the control group. The impedance profiles of HeLa cells treated by distinct dose-dependent drugs were monitored per hour at the optimal frequency. The measured responses were characterized by the cell index (CI) using the following expression [28]:

(2) $$CI=\frac{Z_{\mathrm{cell}}-Z_{\mathrm{cell}-\mathrm{free}}}{Z_{\mathrm{cell}-\mathrm{free}}}$$

#### 2.3.3. Cell Viability Assay

HeLa cells were incubated at a concentration of 3 × 10^5^ cells/well and cultured in a 96-well plate for 24 h, followed by the treatment of three chemotherapy drugs for further culture for 24 h. The CCK-8 reagent was added to each well, and cells were then incubated for 4 h. Finally, the absorbance (OD) of each well at 450 nm was determined by an enzyme marker. The measurements were repeated three times.

#### 2.3.4. Immunofluorescence Staining

HeLa cells treated by DDP, DOX, and 5-FU were incubated in confocal Petri culture dishes for 24 h, respectively. First, HeLa cells were fixed with 4% polyformaldehyde for 20 min and washed with PBS three times. Then, cells were permeabilized with 0.5% Triton x-100 and washed with PBS three times again. Subsequently, mouse monoclonal anti-vinculin antibody diluted with PBS (1:200) was added into the cell solutions and maintained overnight at 4 °C. Next, CF 488A-labeled goat anti-mouse IgG secondary antibody diluted with PBS (1:500) was added into the cell solutions and maintained for 1 h. Finally, the nuclei of the HeLa cells were stained with DAPI for 15 min to label the cells. Stained HeLa cells were observed under a confocal microscope (FV-1000, Olympus, Tokyo, Japan), and cells without drug treatment were observed as the control group. Experiments were conducted three times and the results were averaged.

#### 2.3.5. Measurement of the Number of Attached Cells Treated by Drugs

In order to exactly reflect changes in the number of cells attached to the substrate due to the treatment of chemotherapy drugs, drug-treated cells were resuspended and reattached to the substrate and then the unattached cells were gently rinsed to better evaluate the number of attached cells. Treated HeLa cells were resuspended and reattached to the Matrigel-coated culture dish. Matrigel was coated on the 96-well plate at 37 °C for 1 h, then blocked with 1% BSA at 37 °C for 1 h. HeLa cells were treated with DDP, DOX, and 5-FU for 24 h and then digested and counted. Cells were resuspended in serum-free medium at a density of 8 × 10^4^ cells/mL, with 0.1 mL of cell solution added to each well of the prepared 96-well plates and incubated at 37 °C for 1 h. Next, unattached HeLa cells were washed out in each well with PBS. Subsequently, 10 μL of CCK-8 solution was added into each well for 3 h at 37 °C, then the absorbance values of the medium were measured at 450 nm. The measurements were repeated three times.

### 2.4. Statistical Analysis

The experimental results were averaged. Tukey’s significance test was used to evaluate the statistical differences between the different groups. Significant differences of results between two groups were marked by * (*P* < 0.05), ** (*P* < 0.01), and *** (*P* < 0.001).

## 3. Results

### 3.1. Screening of Experimental Conditions for Impedance Measurement

The ECIS measurements reflected changes in the impedance of the electrodes according to AC current over time. 

**Figure 4:**
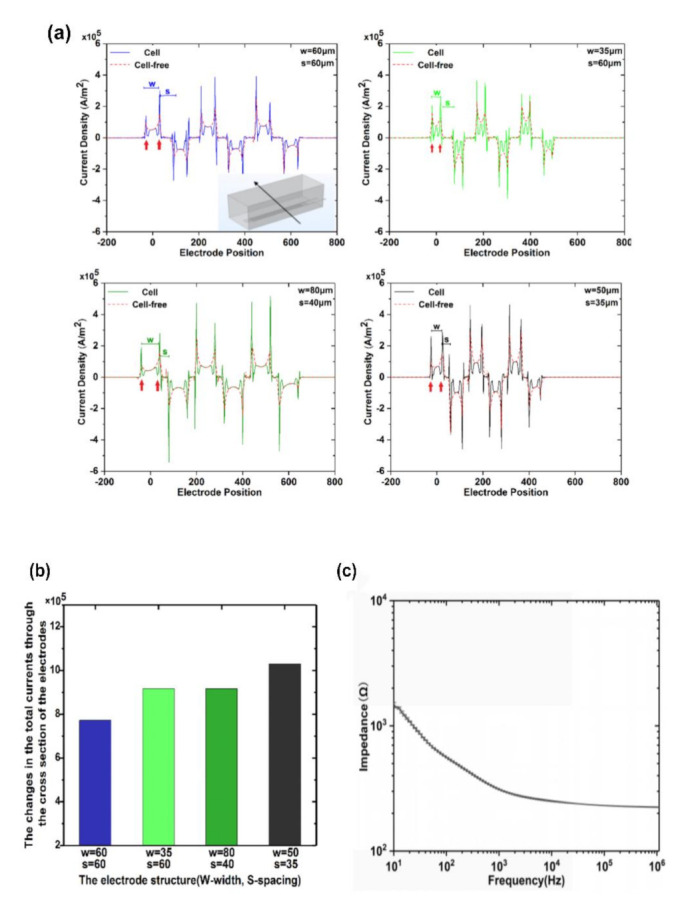
(**a**) Changes in current density passing through the cross section of the electrodes with different dimensions. (**b**) The differences of the total currents through the cross section of the electrodes before and after addition of cells on the top of the electrodes. (**c**) The experimental absolute impedance |Z| value of the selected electrodes, with DMEM as a function of frequency.

Higher current flow promoted sensitivity of the impedance sensor for detection [29]. Poor cell conductivity caused the current to bypass the cell region as the cell grows on the electrodes. Therefore, the distribution of the currents passing through the electrodes could be altered. In FEM simulation, biological cells were added on the top of the electrodes. It was shown that the magnitude and distribution of the current passing through the cross section of the electrodes were altered (Figure 4a). The current density could be reduced where cells are placed due to poor conductivity of cells, as shown by the red arrows in Figure 4a. We integrated the current density along the direction of the black arrow shown in the figure to obtain the total current through the cross section of the electrodes. Cells with the same number were added on the distinct electrodes. Figure 4b exhibits the difference in the integrated current density over the section of the electrodes before and after addition of HeLa cells. The higher difference indicated the electrodes were more sensitive to cell response. A more obvious electrical response of cells could be observed in the electrodes with a finger width of 50 μm and finger–finger spacing of 35 μm in the case of the same input (cells). It was concluded that the electrodes with such structural parameters had higher sensitivity for monitoring changes in cell impedance. Figure 4c shows the experimental absolute impedance |Z| value of the selected electrodes with DMEM as a function of the detection frequency. The electrodes used in this study possessed low base impedance. For instance, the |Z| of the electrodes was around 1.2 kΩ at a frequency of 20 Hz, which was appropriate for conduction of the subsequent experiment using the selected electrodes.

**Figure 5:**
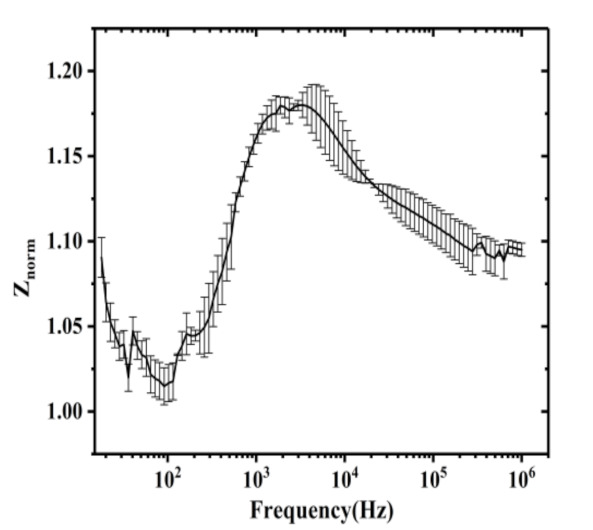
The normalized impedance of the electrodes with HeLa cells as a function of the detection frequency.

Changes in the impedance response of cells could be associated with the sensitivity of the electrodes, the type of cells, and biological processes that cells undergo. It was indicated that the impedance of the electrodes was dependent on the detection frequency. We investigated the optimal detection frequency of the electrodes with HeLa cells. The HeLa cells were incubated on the electrode substrate for 24 h, then the normalized total impedance as a function of the detection frequency was measured, as shown in Figure 5. The experimental results predicted that the maximum normalized total impedance was measured at a frequency of 4 kHz. Therefore, the impedance responses of the HeLa cells to chemotherapy drugs were explored at the optimal frequency.

### 3.2. Real-Time Impedance Response of HeLa Cells to Chemotherapy Drugs

**Figure 6:**
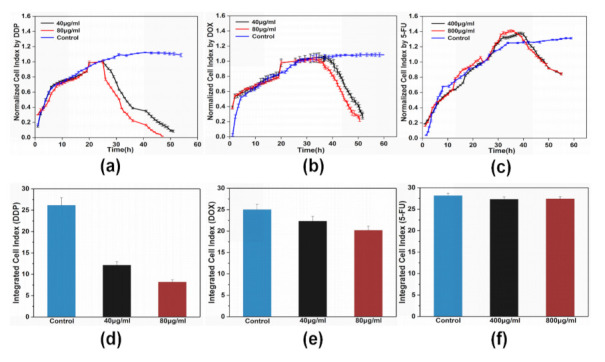
Real-time impedance profiles of HeLa cells treated with (**a**) 40 and 80 µg/mL cisplatin (DDP), (**b**) 40 and 80 µg/mL doxorubicin (DOX), and (**c**) 400 and 800 µg/mL 5-fluorouracil (5-FU). Chemotherapy drugs were added at 24 h. (**d**–**f**) Time-integrated cell index responses of HeLa cells for drug treatments.

The distinct normalized impedance profiles of HeLa cells responding to the treatment of DDP, DOX, and 5-FU at a frequency of 4 kHz and a current of 1.05 mA are shown in Figure 6a–c. The cell indexes of drug-treated cells and their control groups at different time points were normalized by taking the value of cell index of the drug-treated groups at the 24th hour as a baseline, respectively. The normalization method was derived from previous studies [30,31,32,33]. Over the first five hours, HeLa cells rapidly attached to the substrate and the increase of attached cells on the electrodes promoted the increase of the cell index rather than cells outside the electrode. From the 5th hour to the 24th hour, the cell index continued to increase at a slower rate compared to the initial stage. Increasing numbers of cells could have attached to the substrate, and attached cells were probably randomly distributed on or outside the electrodes. After 24 h of cell growth, DDP, DOX, and 5-FU at effective doses were added into the cell samples, respectively. The time points at which the cell index started to decrease were dependent on the type of chemotherapy drug and dose. The cell indexes of DDP-treated HeLa cells began to decline at the 27th hour and the 25th hour at concentrations of 40 and 80 µg/mL, respectively. When HeLa cells were treated with 40 and 80 µg/mL DOX, the decreases in cell indexes occurred at the 38th hour and the 35th hour. Cell indexes of HeLa cells treated with 5-FU continued to increase at a very slow rate until about 16 hours after the drug was added, meaning that 5-FU-treated cells at doses of 400 and 800 µg/mL exhibited decreased cell indexes at the 43rd hour and the 40th hour, respectively. Cell indexes of the DDP-treated HeLa cells declined rapidly between the 25th hour and the 35th hour, then at a slower rate after the 35th hour. Treatment with DOX decreased the cell index rapidly and in a linear fashion. Cell indexes of 5-FU-treated HeLa cells decreased gradually at a slow rate. The time-integrated cell index responses represent an integration of the area under the cell index versus time curve, as shown in Figure 6d–f. The time-integrated impedance output of HeLa cells was dependent on the amplitude and duration of the cell index and could be characterized as a marker to evaluate drug efficacy. The responses were integrated from the 24th hour to the 48th hour. It was observed that HeLa cells were more sensitive to DDP than DOX and 5-FU, with further increases in drug concentrations seeming to have limited effect on the impedance responses of HeLa cells to drug treatment. The HeLa cells exhibited an obviously delayed response to 5-FU treatment, with the cell index continuing to increase from the 24th hour to the 36th hour (800 µg/mL) and the 42nd hour (800 µg/mL), therefore, the integrated cell indexes of the 5-FU-treated HeLa cells were similar.

**Figure 7:**
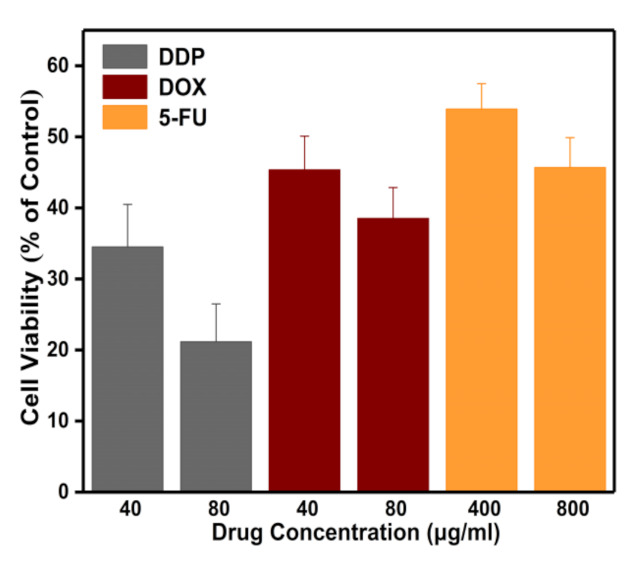
Viability of HeLa cells by dose-dependent DDP, DOX, and 5-FU for 24 h. Cell viability is represented by the ratio of the absorbance (OD) values of cells treated with drugs to the OD values of the control group.

Measurements of cell viability were performed to explore distinct dose-dependent percentages of viable HeLa cells treated with DDP, DOX, and 5-FU for 24 h, respectively, as shown in Figure 7. DDP at an effective dose imposed the most obvious inhibitory effect on the viability of HeLa cells, followed by DOX, with 5-FU exhibiting the lowest inhibitory function. The ratios of viable HeLa cells treated with 40 and 80 µg/mL DDP for 24 h to those without drug treatment were 34% and 21%, respectively. The respective cell indexes of DDP-treated HeLa cells with the abovementioned concentrations at 48 h (corresponding to 24 h treatment of DDP) were around 25% and 14% of the control group. The impedance responses of the HeLa cells treated with DDP therefore appeared to be stronger than in the CCK8-based viability assay. A similar trend was observed for DOX-and 5-FU-treated HeLa cells, indicating that impedance-based cell responses are associated with cell viability, cell adherence, and other factors [34]. Cell viability tests were performed 24 h after treatment of drugs, as indicated in Figure 7, probably including some cells samples that were still alive but not in good condition (e.g., poor adhesion to the substrate). In the following section, analysis of the cell attachment investigations is discussed. 

### 3.3. Changes in Distribution and Intensity of Vinculin after Treatment with Chemotherapy Drugs

**Figure 8:**
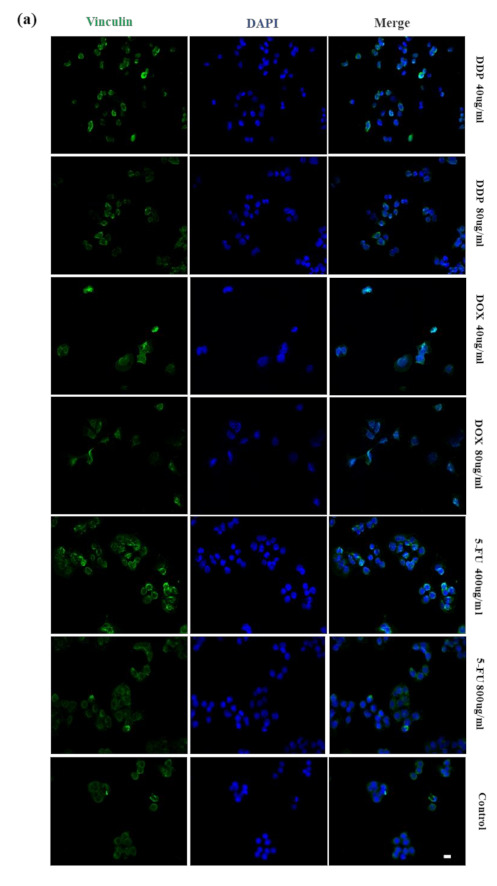

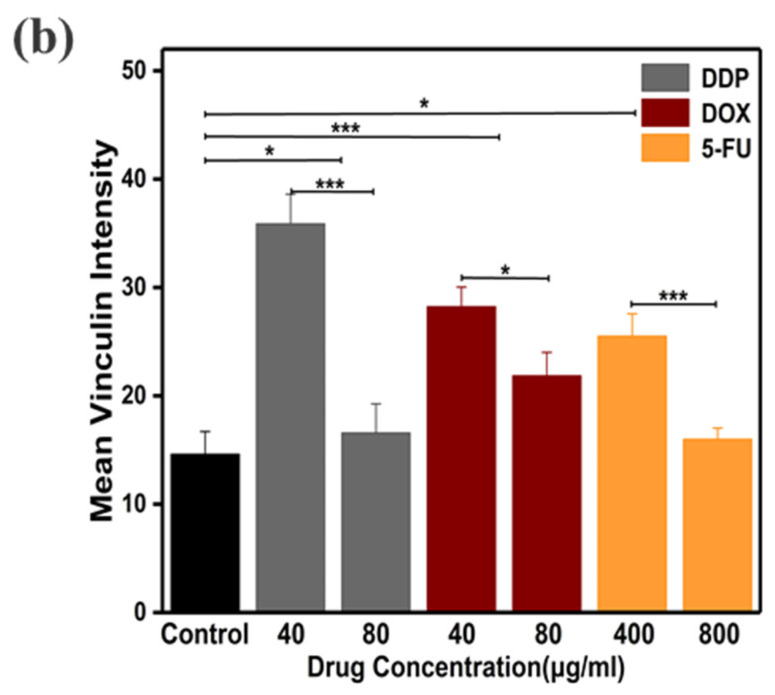
(**a**) Distribution of vinculin of HeLa cells treated by dose-dependent DDP, DOX, and 5-FU for 24 h; vinculin (green), DAPI (blue). (**b**) Average fluorescence intensity of vinculin. Scale bar: 20 µm.

Many studies demonstrated that chemotherapy drugs regulated the expression of adhesion-related proteins [35,36]. Vinculin, which directly binds to the cytoskeleton of cells, is one of the crucial components of focal adhesion (FA). Expression of vinculin was previously indicated to mediate cell adhesion and cell migration [37,38,39]. The distance between cells and the substrate was extended due to a decrease in vinculin level, with more current flow passing through the gap between the cells and the electrodes. The correlation between the impedance responses of the cells and the attachment of cells was investigated. Figure 8a exhibits the dose-dependent distributions of the vinculin of the HeLa cells after 24 h of treatment with DDP, DOX, and 5-FU, respectively. Compared with the control group, the vinculin of HeLa cells treated with DDP at concentrations of 40 and 80 µg/mL were more obviously distributed on the periphery of cells, particularly in the treatment of 40 µg/mL DDP. A similar trend was also observed in HeLa cells treated with 400 and 800 µg/mL 5-FU, respectively. Vinculin of HeLa cells treated with 40 µg/mL DOX was almost evenly distributed within the cells. Distribution of vinculin under the treatment of 80 µg/mL DOX was similar to that in the control group. Changes in the expression of vinculin of HeLa cells are characterized by the fluorescence intensity of vinculin. The mean intensity of vinculin is shown in Figure 8b. The intensity of the vinculin in the HeLa cells was significantly enhanced after treatment with DDP, DOX, and 5-FU, particularly at lower concentrations, demonstrating that the tumor microenvironment provided a refuge for the tumor cells, which reduced their sensitivity to chemotherapy drugs by changing their adhesion behavior [40,41]. Thus, the upregulated expression of vinculin of HeLa cells treated with lower concentrations could be performed to resist drug toxicity. High-dose drug toxicity could outstrip the ability of HeLa cells to resist, resulting in deceased vinculin expression [42]. High expression of vinculin was shown to promote the stabilization of focal adhesion and inhibit cell migration [43,44]. We therefore assume that high expression of vinculin of HeLa cells treated with chemotherapy drugs could stabilize HeLa cells.

### 3.4. Changes in the Number of Attached HeLa Cells Treated with Chemotherapy Drugs

**Figure 9:**
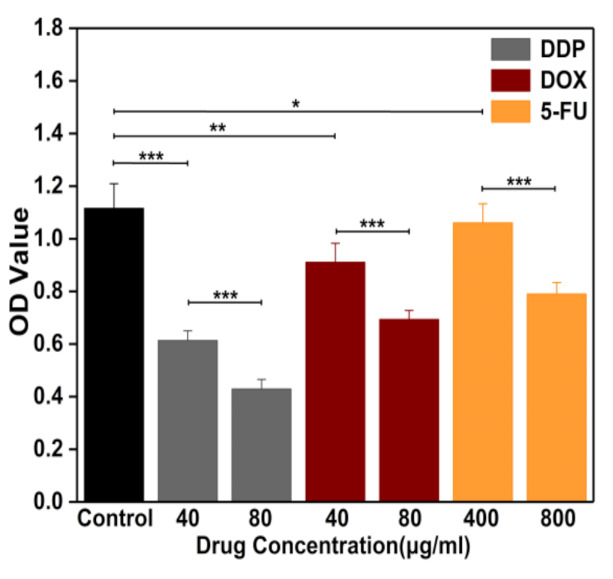
Proliferation of HeLa cells treated dose-dependently with DDP, DOX, and 5-FU based on the CCK-8 assay.

The impedance responses of the HeLa cells to the chemotherapy drugs were closely associated with cells cultured on the surfaces of the electrodes. Attached HeLa cells were measured after treatment with DDP, DOX, and 5-FU, respectively, for 24 h. Figure 9 indicates that all three chemotherapy drugs with increased concentrations showed corresponding decreases in the numbers of attached HeLa cells. A minimal number of DDP-treated HeLa cells is shown compared with the number of cells treated by DOX and 5-FU. Based on the principle of impedance measurement, cells on the surfaces of the electrodes could disrupt the electrode–electrolyte interface and further regulate the whole impedance of the system. Inhibition of the proliferation of drug-treated HeLa cells grown on the electrodes could result in lack of cell contact with the electrodes.

**Figure 10:**
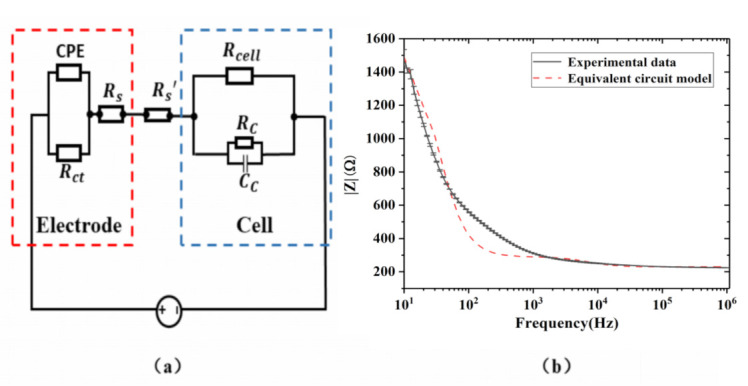
(**a**) Equivalent circuit model of the cell–electrode impedance test system. (**b**) Equivalent circuit fitting result.

ECIS-based measurement detected changes in the impedance of the electrodes to AC current flow over time. When cells were cultured on the surface of the electrodes, the area of the electrodes was blocked, forcing the current on the electrode/electrolyte interface to flow through the cell bodies and cell–cell gaps. Given alterations in the behavior of the attached HeLa cells treated with DDP, DOX, and 5-FU as mentioned above, the cell–electrode electrical circuit model was utilized to extract the electrical properties of the drug-treated Hela cells. It was demonstrated that the currents could pass through cells, cell–cell gaps, and cell–substrate gaps in the process of ECIS testing [45]. Additionally, it was indicated that the current would flow through the cells after flowing through the cell–substrate gaps [46]. The model exhibited biological cell electrical behavior in series with the electrodes, as shown in Figure 10a. The electrical behaviors of the electrodes and cells are represented by Randles’s circuit with a Warburg element and RC circuit [47,48]. CPE was constant phase element, *R_ct_* denoted charge transfer resistance, and *Rs* represented medium resistance. This circuit model establishes the link between the electrical properties of the cells and their biological functions. The impedance responses of the HeLa cells reflected integrated drug efficacy, providing a potential marker of drug efficacy evaluation. The increase in cell number decreased the intercellular distances, leading to less current flow passing through the intercellular distances. On the other hand, larger intercellular distances caused by the inhibition of cell proliferation increased the current flow passing through the intercellular distances. In our circuit model, the resistance, *R_cell_*, reflected changes in the current flow passing intercellularly. Therefore, changes in the numbers of cells treated with chemotherapy drugs were characterized by the resistance *R_cell_*. Higher *R_cell_* values represented cell proliferation. Based on this study, the expression levels of vinculin, an important focal adhesion protein, of cells treated with chemotherapy drugs were altered, indicating changes in adhesion between cells and the substrate regardless of the region, for example, the electrode region or the area between the electrodes. Hence, the distance between the cells and the substrate could be extended due to decreased vinculin, with more current flow passing through the gaps between cells and the electrodes. The resistance, *R′_s_*, reflected these changes in the current flow passing through these regions. Higher values of *R′_s_* represented higher cell–electrode adhesion [49].

We further investigated the correlation between impedance responses and cell–cell or cell–electrode contact of HeLa cells treated with dose-dependent DDP, DOX, and 5-FU at 48 h, corresponding to 24 h of drug treatment. The values of each electrical element of the electrodes were derived by fitting with the experimental impedance–frequency curve of the electrodes without cells cultured on them, shown in Figure 4c, using ZView software. The experimental results were well fitted. The electrical responses of cells were simply described by RC circuit, which was commonly used in other studies [35]. The conductivity and relative dielectric constant of cells were 1 × 10^−5^ (S/m) and 10, respectively, and the thickness of the cell membrane was 0.007 µm [50]. The intrinsic electrical parameters of the electrodes were not affected by the addition of cells. The model was validated based on total impedance experimental after 24 h of growth without drug treatment as a function of sweeping frequency, as shown in Figure 10b. In our model, it is assumed that *R′_s_* and *R_cell_* were proportional to the strength of the cell–substrate adhesion and cell count regulated by chemotherapy drugs. The strength of the cell–substrate adhesion was characterized by the fluorescence intensity of vinculin (*F*), and the cell count was denoted by the optical density (*OD*) resulting from the CCK-8 experiments. Therefore, the total impedance of the cell–electrode system was approximately expressed as follows:

(3) $$z_{total}=R_{s}\prime +R_{cell}+z_{electrode}$$

(4) $$z_{total}=k_{1}F+k_{2}OD+z_{electrode}$$

Based on the method of the multiple linear regression, the fitted proportionality coefficients, k1 and k2, were 2.72 ± 0.14 and 109.56 ± 3.31. Therefore, *R′_s_* and *R_cell_* responding to the simulation of dose-dependent DDP, DOX, and 5-FU for 24 h were shown in Table 1. The model results predicted that cell–cell contact of HeLa cells treated with increased concentrations of DDP, DOX, and 5-FU would be lower compared with the control group, and higher cell–electrode contact of HeLa cells treated with low-dose drugs would be more obvious than HeLa cells treated with high-dose drugs. Therefore, the impedance profiles of HeLa cells responding to the treatment of DDP, DOX, and 5-FU could be regulated by combining the effects of cell–cell and proliferation-related cell–electrode contact.

## 4. Conclusions

In this study, Au interdigitated electrodes were utilized to investigate the impedance profiles of HeLa cells treated with three model drugs (DDP, DOX, and 5-FU). The distinct impedance responses of HeLa cells to different doses of chemotherapy drugs were exhibited. Time-integrated impedance cell index values under different drug treatment stimulations reflected HeLa cell responses under drug treatment. Changes in impedance responses were related with the combination of vinculin-mediated cell adhesion to the substrate and the cell proliferation rate. The values of the adhesion-related resistance and the proliferation-related resistance could be derived from the circuit model based on the method of multilinear regression to demonstrate the distinct weighted roles of cell adhesion and proliferation in impedance responses of HeLa cells under the treatment of DDP, DOX, and 5-FU. The proposed cell index normalization and integration adds new understanding to impedance measurements to more comprehensively reflect the behavioral responses of cells to various stimuli. Our study could facilitate the scientists and clinicians to select more effective schemes for the treatment of the cervical cancer and even other types of cancers based on the specific impedance responses.

## Figures and Tables

**Table 1:** Derived *R′_s_* and *R_cell_* of HeLa cells treated dose-dependently with DDP, DOX, and 5-FU.

R’_s_ (Ω)	41.58 ± 3.71		96.43 ± 3.86	47.79 ± 3.99	76.31 ± 4.19	59.37 ± 2.7	68.83 ±4.15	42.49 ± 1.35
R_cell_ (Ω)	124.74 ± 1.74		65.54 ± 3.01	45.81 ± 2.52	98.73 ± 3.53	77.47 ± 1.27	113.37 ± 5.16	89.82 ± 0.05
